# Outcomes of Sodium-Glucose Cotransporter 2 Inhibitor Use in Adults With Congenital Heart Disease

**DOI:** 10.1016/j.cjcpc.2024.02.001

**Published:** 2024-02-17

**Authors:** Snigdha Karnakoti, Kartik Andi, William R. Miranda, C. Charles Jain, Luke J. Burchill, Maan Jokhadar, Heidi M. Connolly, Alexander C. Egbe

**Affiliations:** Department of Cardiovascular Medicine, Mayo Clinic Rochester, Rochester, Minnesota, USA

**To the Editor:**

The sodium-glucose cotransporter 2 inhibitor (SGLT2i) is one of the cornerstones of guideline-directed medical therapy for heart failure and has been shown to reduce the risk of mortality and heart failure hospitalization, and improve functional status and quality of life in patients with heart failure.^1^ However, there are limited data about the safety and clinical benefits (or lack thereof) of SGLT2i for the management of heart failure in adults with congenital heart disease (CHD), even though heart failure is the leading cause of mortality in this population.^2^^,^^3^ The purpose of this study was to describe the outcomes and safety of SGLT2i in adults with CHD and heart failure.

We identified adults with CHD and heart failure who received SGLT2i (2018-2022) for >12 months without surgical or transcatheter intervention in the course of SGLT2i therapy. The primary outcome was improvement in functional status defined as change in (Δ) New York Heart Association (NYHA) class. The secondary outcomes were (1) Δ N-terminal prohormone brain natriuretic peptide (NT-proBNP), (2) Δ estimated glomerular filtration rate (eGFR), (3) Δ model for end-stage liver disease excluding international normalized ratio (MELD-XI) score, and (4) Δ systemic ventricular global longitudinal strain based on offline image analysis. See Supplemental Appendix S1 for details of patient selection and CHD diagnosis.

Of 437 adult patients with CHD and heart failure, 24 (6%) met the study inclusion criteria (age 54 ± 13 years, 17 men [71%], systemic left ventricle [LV] 19 [79%], and diabetes 21 [88%]; Table 1).

**Table 1:** Baseline characteristics

Characteristics	Baseline (n = 24)	Follow-up (n = 24)	*P*
Age (y)	54 ± 13	55 ± 11	
Male, n (%)	17 (71)	17 (71)	
Systemic left ventricle, n (%)	19 (79)	19 (79)	
Systolic blood pressure (mm Hg)	129 ± 11	122 ± 14	0.3
Weight (kg)	89 ± 17	81 ± 14	0.1
Body mass index (kg/m^2^)	28.6 ± 3.1	28.2 ± 2.9	0.4
Comorbidities, n (%)			
Hypertension	20 (83)	20 (83)	0.9
Diabetes	21 (88)	21 (88)	0.9
Coronary artery disease	13 (54)	13 (54)	0.9
Medications, n (%)			
β-Blockers	24 (100)	24 (100)	0.9
ACEI/ARB	22 (92)	22 (92)	0.9
ARNI	2 (8)	2 (8)	0.9
Mineralocorticoid receptor antagonist	14 (58)	14 (58)	0.9
Loop diuretics	20 (83)	18 (75)	0.6
Laboratory data			
eGFR (mL/min/1.73 m^2^)	74 (63-101)	88 (69-124)	0.009
NT-proBNP (pg/mL)	803 (416-1585)	611 (309-1214)	0.006
MELD-XI score	10.6 (9.7-12.1)	10.1 (9.4-11.4)	0.08
Fasting glucose (mg/dL)	98 ± 8	91 ± 6	0.2
Haemoglobin A1C	7.2 ± 1.2	6.5 ± 0.9	0.1
NYHA, n (%)			<0.001
I	0	9 (38)	
II	10 (42)	9 (38)	
III	10 (42)	4 (17)	
IV	4 (17)	2 (8)	
Echocardiogram (systemic LV)			
Patients with systemic RV, n (%)	19 (79)		
Systemic LV GLS (%)	−15 ± 3	−17 ± 4	0.07
Systemic LV EF (%)	44 ± 11	47 ± 10	0.2
Nonsystemic RV GLS (%)	−22 ± 5	−25 ± 5	0.1
Nonsystemic RV FAC (%)	38 ± 7	41 ± 10	0.3
Patients with systemic RV, n (%)	5 (21)		
Systemic RV GLS (%)	−12 ± 3	−14 ± 4	0.2
Systemic RV FAC (%)	26 ± 9	28 ± 10	0.5
Nonsystemic LV GLS (%)	−17 ± 4	−16 ± 4	0.8
Nonsystemic LV EF (%)	58 ± 11	55 ± 9	0.7

ACEI/ARB, angiotensin-converting enzyme inhibitor/angiotensin-II receptor blocker; ARNI, angiotensin receptor neprilysin inhibitor; EF, ejection fraction; eGFR, estimated glomerular filtration rate; FAC, fractional area change; GLS, global longitudinal strain; LV, left ventricle; MELD-XI, model for end-stage liver disease excluding international normalized ratio; NT-proBNP, N-terminal prohormone brain natriuretic peptide; NYHA, New York Heart Association; RV, right ventricle.

The following SGLT2i agents were used: empagliflozin 10 mg (n = 10, 41%), empagliflozin 25 mg (n = 5, 21%), and dapagliflozin 10 mg (n = 9, 38%). The median interval between baseline and follow-up assessments (ie, duration of therapy) was 19 (14-31) months. Compared with baseline indices, we observed a temporal reduction in the proportion of patients who were symptomatic (NYHA III/IV n = 14 [58%] vs NYHA III/IV n = 6 [25%], *P* = 0.01) and a temporal improvement in neurohormonal activation (ΔNT-proBNP −192 [95% confidence interval (CI): −311 to −105] pg/mL, *P* = 0.006) and renal function (ΔeGFR 14 [6-22] mL/min/1.73 m^2^, *P* = 0.09). However, there was no significant change in hepatorenal function (ΔMELD-XI score) or systemic ventricular function (Table 1).

Compared with patients with systemic right ventricle (n = 5, 21%), those with systemic LV (n = 19, 79%) had a similar temporal change in outcomes: ΔNT proBNP (−216 [95% CI: −283 to −141] vs −181 [95% CI: −273 to −94], *P* = 0.1) and ΔeGFR (16 [11-22] vs 13 [8-19], *P* = 0.4). Compared with patients with systemic LV ejection fraction <50% (n = 13, 54%), those with systemic LV ejection fraction ≥50% (n = 6, 25%) had a similar temporal change in outcomes: ΔNT proBNP (−198 [95% CI: −304 to −122] vs −235 [95% CI: −298 to −101], *P* = 0.3) and ΔeGFR (12 [7-19] vs 15 [9-22], *P* = 0.3). With regards to safety end points, there was no renal adverse event, fasting hypoglycaemia, or major hypoglycaemic event.

In conclusion, SGLT2i, used in conjunction with other medical therapy for heart failure, was associated with improvement in functional status, neurohormonal activation, and renal function.
