# Supplementary material for: Outcomes of Sodium-Glucose Cotransporter 2 Inhibitor Use in Adults With Congenital Heart Disease
Source: CJC Pediatr Congenit Heart Dis. 2024 Feb 17;3(3):115–6. doi: 10.1016/j.cjcpc.2024.02.001 (PMC11282876; doi:10.1016/j.cjcpc.2024.02.001)
Supplement: Supplemental Appendix S1 [file mmc1.docx]

**SUPPLEMENTARY DATA**

**METHODS**

We identified adults with CHD and heart failure that received SGLT2i (2018-2022) for >12 months without surgical or transcatheter intervention in the course of SGLT2i therapy. The clinical indices collected within 1 month prior to the initiation of SGLT2i were used to define the clinical characteristics of the cohort. Temporal changes in outcomes were assessed between 12-36 months after initiation of SGLT2i. The primary outcome was improvement in functional status defined as change in (∆) New York Heart Association (NYHA) class. The secondary outcomes were: (1) ∆ N terminal pro hormone brain natriuretic peptide (NT-proBNP), (2) ∆ estimated glomerular filtration rate (eGFR), (3) ∆ model for end-stage liver disease excluding international normalized ratio (MELD-XI) score, (4) ∆ systemic ventricular global longitudinal strain (GLS) based on offline image analysis. Pre-specified subgroup analyses were performed to compare outcomes in patients with: (1) systemic left ventricle (LV) versus systemic right ventricle; (2) systemic LV ejection fraction (EF) <50% versus ≥50%, (3) diabetes versus no diabetes.

The safety endpoints were: (1) renal adverse event defined as >50% reduction in eGFR from baseline; (2) fasting hypoglycemia defined as fasting blood sugar <70 mg/dl; (3) major hypoglycemic event defined as hypoglycemia requiring the assistance of another person to actively administer carbohydrates or glucagon or to take other corrective action.

Statistical analysis

Data were presented as mean ± standard deviation, median (interquartile range), and count (%) for continuous variable with normal distribution, continuous variable with skewed distribution, and categorical variables, respectively. Between-group comparisons were performed with unpaired t test for continuous variable with normal distribution, and Wilcoxon rank sum test for continuous variable with skewed distribution. Temporal improvements in outcomes were assessed using unpaired t test for continuous variable, and McNemar’s test for categorical variables. All statistical analyses were performed with BlueSky Statistics software (version. 7.10; BlueSky Statistics LLC, Chicago, IL, USA), P value <0.05 was considered to be statistically significant for all analyses

RESULTS

Out of 437 adults with CHD and heart failure, 24 (6%) patients that met study inclusion criteria (age 54±13 years, 17 men [71%], and diabetes 21 [88%]). The CHD diagnoses were: Fontan palliation (n=1), tetralogy of Fallot (n=5), coarctation of aorta (n=4), congenitally corrected transposition of great arteries (n=2), d-transposition of great arteries status post atrial switch operation (n=3), truncus arteriosus (n=1), d-transposition of great arteries status post arterial switch operation (n=2), congenital aortic stenosis (n=1), atrioventricular canal defect (n=1), and atrial septal defect (n=4). Of the 24 patients, 23 (96%) add biventricular circulation, and 19 (79%) had systemic LV.
